# Transforming Anesthesia Data Into the Observational Medical Outcomes Partnership Common Data Model: Development and Usability Study

**DOI:** 10.2196/29259

**Published:** 2021-10-29

**Authors:** Antoine Lamer, Osama Abou-Arab, Alexandre Bourgeois, Adrien Parrot, Benjamin Popoff, Jean-Baptiste Beuscart, Benoît Tavernier, Mouhamed Djahoum Moussa

**Affiliations:** 1 Univ. Lille CHU Lille ULR 2694 - METRICS: Évaluation des technologies de santé et des pratiques médicales Lille France; 2 InterHop Paris France; 3 Univ. Lille Faculté Ingénierie et Management de la Santé Lille France; 4 Department of Anaesthesiology and Critical Care Medicine Amiens Picardie University Hospital Amiens France; 5 Department of Anesthesiology and Critical Care Medicine Regional University Hospital of Nancy Nancy France; 6 Department of Anaesthesiology and Critical Care Rouen University Hospital Rouen France; 7 Department of Anesthesiology and Critical Care CHU Lille Lille France

**Keywords:** data reuse, common data model, Observational Medical Outcomes Partnership, anesthesia, data warehouse, reproducible research

## Abstract

**Background:**

Electronic health records (EHRs, such as those created by an anesthesia management system) generate a large amount of data that can notably be reused for clinical audits and scientific research. The sharing of these data and tools is generally affected by the lack of system interoperability. To overcome these issues, Observational Health Data Sciences and Informatics (OHDSI) developed the Observational Medical Outcomes Partnership (OMOP) common data model (CDM) to standardize EHR data and promote large-scale observational and longitudinal research. Anesthesia data have not previously been mapped into the OMOP CDM.

**Objective:**

The primary objective was to transform anesthesia data into the OMOP CDM. The secondary objective was to provide vocabularies, queries, and dashboards that might promote the exploitation and sharing of anesthesia data through the CDM.

**Methods:**

Using our local anesthesia data warehouse, a group of 5 experts from 5 different medical centers identified local concepts related to anesthesia. The concepts were then matched with standard concepts in the OHDSI vocabularies. We performed structural mapping between the design of our local anesthesia data warehouse and the OMOP CDM tables and fields. To validate the implementation of anesthesia data into the OMOP CDM, we developed a set of queries and dashboards.

**Results:**

We identified 522 concepts related to anesthesia care. They were classified as demographics, units, measurements, operating room steps, drugs, periods of interest, and features. After semantic mapping, 353 (67.7%) of these anesthesia concepts were mapped to OHDSI concepts. Further, 169 (32.3%) concepts related to periods and features were added to the OHDSI vocabularies. Then, 8 OMOP CDM tables were implemented with anesthesia data and 2 new tables (EPISODE and FEATURE) were added to store secondarily computed data. We integrated data from 5,72,609 operations and provided the code for a set of 8 queries and 4 dashboards related to anesthesia care.

**Conclusions:**

Generic data concerning demographics, drugs, units, measurements, and operating room steps were already available in OHDSI vocabularies. However, most of the intraoperative concepts (the duration of specific steps, an episode of hypotension, etc) were not present in OHDSI vocabularies. The OMOP mapping provided here enables anesthesia data reuse.

## Introduction

Observational health data collected from electronic health records (EHRs) can be valuable not only for direct health care delivery but also for secondary uses (ie, data reuse) in research, evaluating quality of care, and public health [1,2]. Concerns on data reuse include data validity and lack of reproducibility [3-5]. These concerns have driven the need for a framework to enhance the secondary use of health data [6]. To support reproducible research over a distributed research network, Observational Health Data Sciences and Informatics (OHDSI) provides the Observational Medical Outcomes Partnership (OMOP) common data model (CDM) and a full range of open-source tools and methods [7-12]. OHDSI provides database scripts for implementing the CDM on various database systems, a terminology browser to navigate through vocabularies integrated into the OMOP CDM (Athena), a data quality tool used to characterize and visualize a database’s conformity with the OMOP CDM (Achilles), methods for connecting to the OMOP CDM (DatabaseConnector), methods for the extract-transform-load process (WhiteRabbit, RabbitInAHat, and Usagi), methods for data extraction and transformation (OhdsiRTools and FeatureExtraction), and methods for statistical analyses and machine learning (PatientLevelPrediction, CohortMethod, CaseCrossover, and CaseControl) [13-15].

The OMOP CDM standardizes the vocabulary and structure of EHRs and medical claims data to promote interoperability and ensure that queries can be applied consistently to distributed databases. Integration of local data into the CDM involves conceptual mapping of local concepts into standard vocabulary concepts and structural mapping of local entities to standard entities in the OMOP CDM [8,16]. The essential conceptual and structural mapping of local data is time- and resource-consuming and may also result in the loss of information [11]. However, once mapped, the data offer new opportunities [8,11]. In 2020, more than 100 databases from 20 countries (corresponding to more than 0.5 billion patients) have been integrated into the OMOP CDM [12]. Most of the data come from claims databases studied for pharmacoepidemiological purposes [17-21] or from hospital clinical databases [22,23]. In the past decade, many studies have been carried out; they include patient-level predictions and estimations of the population-level effect [24-27]. Recently, Lane et al collected data on 9,00,000 patients in 15 centers using different software packages; this highlights opportunities for collaboration between centers and for increasing the power of such studies [28].

Even though many studies have been published, some aspects of integrating data into the OMOP CDM are still challenging. Cho et al showed that semantic mapping of concepts from organ transplantation registry forms was fastidious and that OMOP concepts covered only 55% of their vocabulary [29]. Michael et al mapped only 26% of local biospecimen records to the OMOP CDM owing to missing information [30]. Researchers have suggested adapting the CDM (by adding new concepts or new fields) to support the integration of biospecimen data. Warner et al added an extension to the OMOP CDM to support cancer treatments and handle episodes of care with a higher level of abstraction than that represented in the OMOP tables of low-level clinical events [31].

In the field of intraoperative management and anesthesiology, several retrospective studies have looked for links between hemodynamic variations (eg, hypotension) in the operating theater and negative postoperative outcomes (eg, death and acute kidney injury) [32-34]. Similar results were observed for the intraoperative tidal volume ventilation administered to patients [35]. In several cases, this work has made it possible to generate hypotheses for prospective studies, the results of which then validated the proposed hypotheses [36]. These studies were mainly performed with data automatically collected by anesthesia information management systems (AIMS) [37]. However, most of the studies were performed at a small number of centers, which reduced the results’ external validity. The main specific features of data recorded in the operating room are their high frequency and high degree of precision, with 1 data point saved every 30 seconds for signals like the heart rate or the intra-arterial blood pressure. Another specific feature is the ability to transform raw data into more usable information or new variables that may better describe exposure to an insult. For example, the arterial pressure signal is computed into comprehensive hypotension events, including the number of episodes, area under the curve, and average time spent within or beyond a threshold [38,39]. In terms of anesthesia data, these data warehouse–based studies can be potentially extrapolated to an international dimension, with stronger evidence through data sharing. This sharing requires the prior homogenization of vocabularies, data formats, and data quality, as promoted by OMOP. However, anesthesia data have not previously been mapped into the OMOP CDM, and the proportion of the anesthesia vocabulary that has already been mapped has not been determined.

The primary objective of the present study was to standardize anesthesia data to the OMOP CDM. The secondary objectives were to provide vocabularies for the reuse of large-scale data and develop queries and dashboards related to the exploitation of anesthesia data using the OMOP CDM.

## Methods

### Study Data

**Figure 1:**
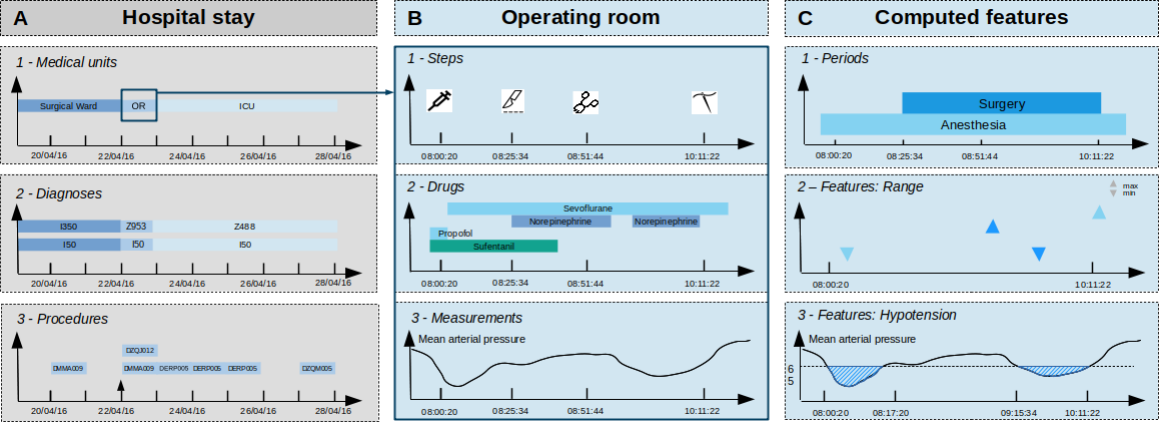
Example of local data organization for a cardiac surgery stay. A. hospital data from the Programme de Médicalisation des Systèmes d’Information database: medical units, diagnoses, and procedures. B. anesthesia information management systems data: steps in the procedures, drug administrations, and measurements. C. features computed from anesthesia information management systems data: periods of interest (anesthesia and surgery), features (range of mean arterial pressure during anesthesia and surgery, and the duration and number of episodes with a mean arterial pressure below 65 mm Hg).

Lille University Medical Center (Lille, France) has developed a clinical data warehouse with a local data model [40]. This data warehouse has been collecting data related to the hospital stay and operating room since 2010. Other features were also subsequently computed to facilitate data reuse. Hence, data were classified into three types, as shown in Figure 1: hospital stay data, operating room data, and computed features.

### Hospital Stay Data

Hospital stay data were extracted from the French national discharge database (Programme de Médicalisation des Systèmes d’Information [PMSI]) used by all hospitals in France and are presented in Figure 1A. The PMSI contains medical discharge reports entered after each hospital visit. The hospital stay data include all the characteristics of a patient’s stay, such as the diagnosis (based on the International Classification of Diseases, 10th edition), medical procedures (based on the Classification Commune des Actes Médicaux), and admission and discharge dates. We have previously implemented the PMSI’s administrative data into the OMOP CDM [20].

**Operating Room Data**

Operating room data were extracted from the hospital’s dedicated AIMS [37] and are presented in Figure 1B. Various modules collect and centralize all the data referring to one case, from the preanesthetic evaluation to discharge from the postanesthesia care unit (PACU). These modules include continuously monitored parameters (eg, heart rate, blood pressure, respiratory rate, and tidal volume), drug administrations, and the main steps in anesthesia and surgery procedures.

### Computed Features

**Figure 2:**
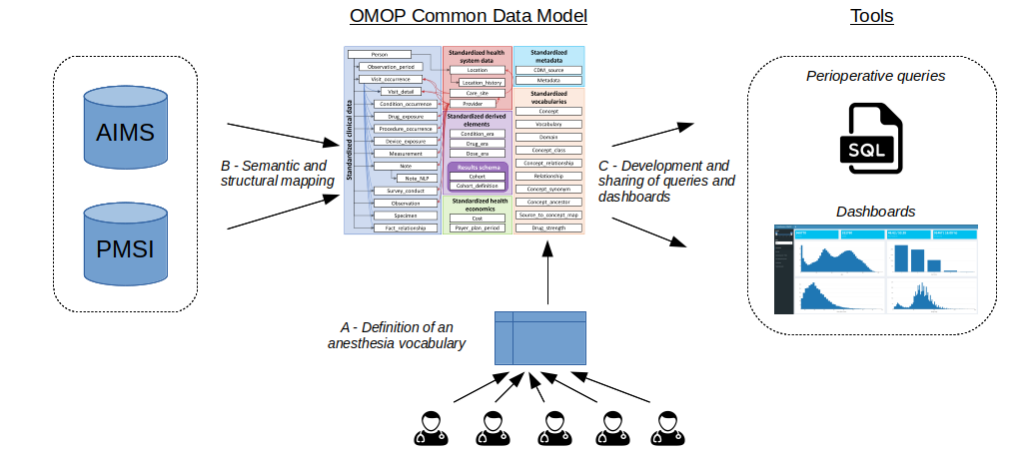
Transformation of anesthesia data into the Observational Medical Outcomes Partnership common data model. A. selection of concepts related to anesthesia procedures by 5 anesthetists. B. semantic and structural mapping of anesthesia and Programme de Médicalisation des Systèmes d’Information data into the Observational Medical Outcomes Partnership common data model. C. development of shareable material for the exploitation of anesthesia data. AIMS: anesthesia information management systems; OMOP: Observational Medical Outcomes Partnership; PACU: postanesthesia care unit; PMSI: Programme de Médicalisation des Systèmes d’Information; SQL: structured query language.

**Figure 3:**
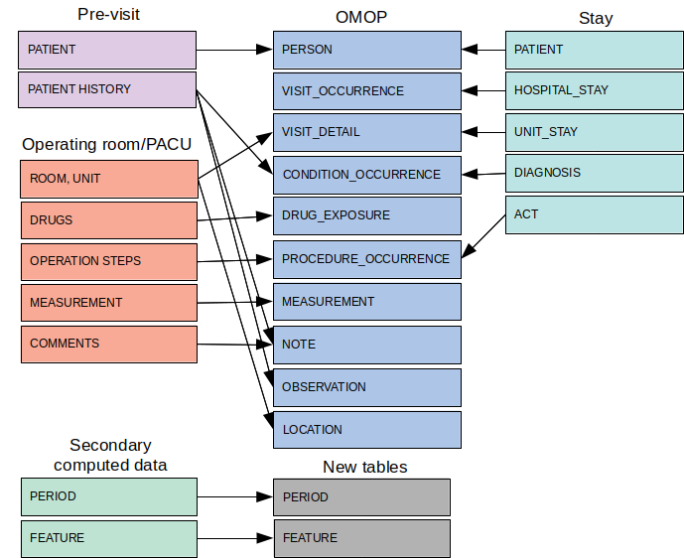
Structural mapping of data related to the preanesthesia consultation and visits to the operating room, and the postanesthesia care unit in the Observational Medical Outcomes Partnership common data model. Integration of secondarily computed data necessitated the implementation of 2 new tables: PERIOD and FEATURE. OMOP: Observational Medical Outcomes Partnership; PACU: postanesthesia care unit.

New features were computed to facilitate data reuse for research purposes [38,39]. First, we determined intraoperative periods of interest from events in time, as shown in Figure 1C-1. Second, we derived perioperative measurements and events from the periods of interest and then specified events (hypotension, tachycardia, and oxygen desaturation) as the ranges, medians, or means, indicated in Figures 1C-2 and 1C-3.

### Semantic and Structural Mapping to the OMOP CDM

The vocabularies used to characterize the patients and anesthesia procedure were identified by 5 experts in anesthesia from 5 different centers (Lille, Amiens, APHP, Nancy, and Rouen) in France. The experts then selected the most relevant concepts for conducting care and research from within these vocabularies. Next, each local concept was mapped to a standard concept from the OHDSI vocabularies, as shown in Figure 2A. Figure 2B shows that structural mapping links the source data table to the OMOP data table and the source columns to the OMOP columns according to the OHDSI specifications [41]. The extract-transform-load process was implemented using a structured query language, and data were stored in a PostgreSQL 10.11 database (PostgreSQL Global Development Group) on Ubuntu 18.04.3.

### Shareable Queries and Dashboards

To test the relevance of the OMOP CDM, we implemented 8 queries related to service audits and clinical research; these were based on the semantic and structural mapping implemented in our database. The queries were intended to provide the following information: (Q1) number of operations per year and per specialty department, (Q2) anesthesia procedures during an outpatient visit, (Q3) operations with fast-track surgery and no admission to the PACU, (Q4) operations with a mean arterial pressure below 65 mm Hg within 30 minutes of anesthesia induction, (Q5) administrations of norepinephrine, epinephrine, ephedrine, phenylephrine, dobutamine, or atropine received within 15 minutes of the first drop in the mean arterial pressure to below 65 mm Hg, (Q6) length of stay according to the score categories of the American society of anesthesiologists, (Q7) operations followed by a stay in the intensive care unit, and (Q8) characterization of the Mallampati grade.

In a previous work, we described the user-centered development, implementation, and preliminary evaluation of clinical dashboards related to anesthesia unit management and quality assessment in the Lille University Medical Center [42]. The user needs had been identified by conducting 21 end-user interviews. Several representations had been developed and submitted to end users for appraisal. After prioritization and feasibility assessment, 10 dashboards were ultimately implemented and deployed. Dashboards were evaluated by 20 end users (4 residents, 4 nurse anesthetists, and 12 anesthesiologists, including the head of the department and a unit manager). The mean (standard deviation) system usability score was 82.6 (11.5), which corresponded to excellent usability. As the dashboards were implemented from our data warehouse with local vocabulary and structured following a local data model, their codes could not be shared with other teams. In the current study, we selected 4 existing dashboards (population description, hemodynamic management, ventilation management, and postoperative outcome) and implemented them from the database now in the OMOP format, as shown in Figure 2C. The dashboards were implemented in R (The R Project for Statistical Computing) with the shiny, shinythemes, shinydashboard, and dplyr packages. The application was connected to the OMOP CDM via the DatabaseConnector package. We compared the new dashboards with the former versions to assess the possible loss of information.

## Results

### Semantic Mapping

The experts identified 8 types of vocabularies that had been custom-developed for the AIMS by software editors and anesthetists or that were used in the data warehouse: patient characteristics on the day of the procedure, types of visits, units, measurements, drugs, operation steps, periods, and features. Patient history–related vocabulary was not considered, as it was mainly documented manually, using synonyms, abbreviations, and negatives. From within the 8 mapped vocabularies, the experts selected the 522 concepts given in Table 1: 23 patient characteristics, 6 visits, 162 drugs, 45 measurement parameters, 67 units, 46 operation steps, 18 periods, and 155 features.

The experts looked for corresponding concepts in the OHDSI standardized vocabularies. Among the 522 concepts, 353 (67.7%) were successfully mapped to standard concepts for patient characteristics, visits, units, measurements, drugs, operation steps, and periods. All the concepts for patient characteristics, units, measurements, operation steps, and drugs were mapped. Further, 169 concepts (32.4%) in the visit, period, and feature vocabularies were not retrieved in the OHDSI standardized vocabularies and were thus added to the CONCEPT table. Specifically, the concept “operating room visit” was a new type of visit and helped distinguish visits to the operating room from the other types of visits in care units (eg, intensive care and emergency units). The semantic mapping is described in Table 1. All the concepts are listed in Multimedia Appendix 1.

Drugs were mapped to standard concepts of the class “ingredient,” as the clinical drug form is not correctly documented in the AIMS.

**Table 1:** Semantic mapping between anesthesia and Observational Health Data Sciences and Informatics vocabularies.

Source vocabularies	Concepts identified in source vocabularies, N	Corresponding standard OHDSI^a^ vocabularies	Concepts mapped to standard OHDSI concepts, n (%)	New concepts added, n
Demographics	23	SNOMED^b^	23 (100)	0
Visits	6	Visit	5 (83.3)	1
Units	67	UCUM^c^/SNOMED	67 (100)	0
Measurements	45	LOINC^d^/SNOMED	45 (100)	0
Operation steps	46	SNOMED	46 (100)	0
Drugs	162	RxNorm	162 (100)	0
Period	18	—^e^	5 (28.8)	13
Feature	155	—	0 (0)	155

^a^OHDSI: Observational Health Data Sciences and Informatics.

^b^SNOMED: Systemized Nomenclature of Medicine.

^c^UCUM: Unified Code for Units of Measure.

^d^LOINC: Logical Observation Identifiers Names and Codes.

^e^Not available.

### Structural Mapping

Each inpatient visit is defined a record in the VISIT_OCCURRENCE table. During a hospital stay, each move to a medical unit or an operating room for an operation is defined as a record in the VISIT_DETAIL table. Operating room visits were characterized with a new “operating room visit” concept, namely VISIT_DETAIL_CONCEPT_ID. This concept made it possible to differentiate between visits to care units and those to the operating room. Diagnoses and medical procedures documented in medical units were linked to the corresponding VISIT_DETAIL and VISIT_OCCURRENCE records. Measurements, drug administrations, and events documented in the operating room or PACU were linked to the corresponding operation by the VISIT_DETAIL_ID. Structural events were mapped to procedure_occurrence. Free-text entries from the preanesthesia consultation and those in the operating room were mapped to NOTE. Owing to the high volume, raw data for continuously monitored variables were not included in the measurement table but were kept aside in another schema. The RELATIONSHIP table was implemented with the relationships between the 214 anesthesia rooms (ie, preanesthesia consultation rooms, operating rooms, and the PACU) and the corresponding specialty departments. Structural mapping of the local clinical tables onto the OMOP tables is described in Figure 3.

**Figure 4:**
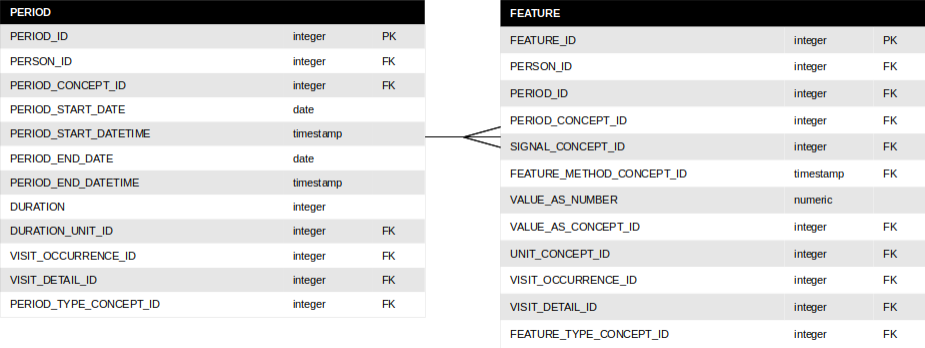
Logical data model of PERIOD and FEATURE tables.

We defined 2 new tables to store the computed data, namely period and feature. A period is defined by 2 milestones, a start event and an end event. The events may come from different sources: administration of a drug, a step in a procedure, consultation with a health care professional, or a visit to a health care unit. A period may be defined by an event date or time and a time interval, such as the start of a procedure and the next 30 minutes, or the administration of a drug and the last 10 minutes. A feature is defined by the combination of three concepts: a period (as defined above), a raw signal, and an aggregation method. The raw signal may include measurements of vital signs (eg, heart rate, arterial pressure, and oxygen saturation) or mechanical ventilation parameters (tidal volume, respiratory rate, and plateau pressure). The aggregation method may be a statistical indicator (eg, the mean, minimum, or maximum value) or an expert-driven rule [35]. The logical data model for these 2 tables is described in Figure 4.

### Integration

Records spanning 10 years were integrated into the OMOP CDM. It corresponded to 5,72,609 operations for 3,29,633 patients. The numbers of records per OMOP table are shown in Table 2, and the number of records per operation and those per hospital stay are given in Table 3.

**Table 2:** Number of records implemented in Observational Medical Outcomes Partnership tables for the 2 data sources.

OMOP^a^ table	Number of records
PERSON	3,29,633
VISIT_OCCURRENCE	48,84,220
VISIT_DETAIL (from PMSI^b^)	15,40,677
VISIT_DETAIL (from AIMS^c^)	5,72,609
CONDITION_OCCURRENCE (from PMSI)	15,13,544
CONDITION_OCCURRENCE (from AIMS)	5,67,442
DRUG_EXPOSURE	86,12,045
PROCEDURE_OCCURRENCE (from PMSI)	11,66,227
PROCEDURE_OCCURRENCE (from AIMS)	5,58,734
OBSERVATION (from PMSI)	18,644
OBSERVATION (from AIMS)	49,45,451
NOTE	92,88,981
PERIOD	40,26,665
FEATURE	3,48,09,015
LOCATION	1,348

^a^OMOP: Observational Medical Outcomes Partnership.

^b^PMSI: Programme de Médicalisation des Systèmes d’Information.

^c^AIMS: anesthesia information management system.

**Table 3:** Median (IQR) number of records per operation and per hospital stay.

OMOP^a^ table	Median (IQR) number of records per operation (AIMS^b^)	Median (IQR) number of records per hospital stay (PMSI^c^)
VISIT_DETAIL	1 (1-1)	1 (1-1)
CONDITION_OCCURRENCE	1 (1-1)	2 (2-5)
DRUG_EXPOSURE	10 (5-17)	—^d^
PROCEDURE_OCCURRENCE	9 (4-12)	2 (1-4)
OBSERVATION	10 (8-10)	—
NOTE	31 (12-40)	—
PERIOD	7 (5-10)	—
FEATURE	71 (42-84)	—

^a^OMOP: Observational Medical Outcomes Partnership.

^b^AIMS: anesthesia information management system.

^c^PMSI: Programme de Médicalisation des Systèmes d’Information.

^d^Not available.

### Shareable Queries and Dashboards

Based on the anesthesia and hospital stay data, we developed 8 queries for application to the existing VISIT_OCCURRENCE, VISIT_DETAIL, CONDITION_OCCURRENCE, PROCEDURE_OCCURRENCE, DRUG_EXPOSURE, NOTE, CONCEPT, and RELATIONSHIP tables and the 2 new PERIOD and FEATURE tables. The query steps and queried tables are described in Table 4. All queries are detailed in Multimedia Appendix 2.

**Figure 5:**
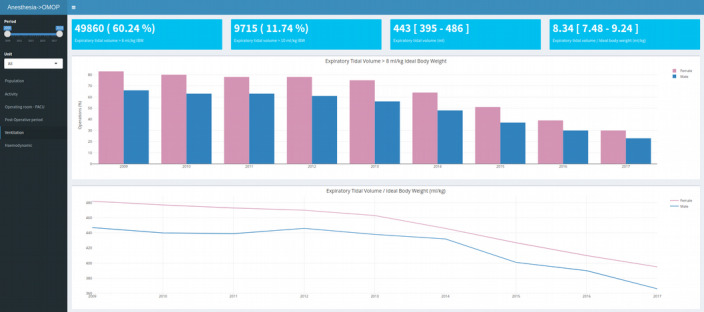
Clinical dashboard for the assessment of ventilatory guidelines. Number of operations with tidal volume>8 ml.kg-1 of ideal body weight, number of operations with tidal volume>10ml.kg-1 of ideal body weight, median (IQR) expiratory tidal volume, median (IQR) expiratory tidal volume/ ideal body weight change over time in the proportion of operations with tidal volume >8 ml.kg-1 of ideal body weight and change over time in expiratory tidal volume/ ideal body weight over the year.

Population description, hemodynamic, ventilation, and postoperative outcome are the 4 dashboards available, as shown in Table 5 and Figure 5. They provide an overview of the population treated in the operating room, compliance with hemodynamic guidelines, compliance with ventilatory guidelines, and postoperative outcomes. Each dashboard can be configured through filtering by year and department. The tables PERSON, VISIT_DETAIL, OBSERVATION, and FEATURE were queried to feed the dashboards. Although the format of the data source differed between the 2 versions of the dashboards (local format vs OMOP format), the figures and results obtained were identical.

**Table 4:** List of queries in the context of the operating room visits and hospital stays.

Query ID	Query	Requirement	Query steps and queried tables
1	Number of operations per year and per specialty department	Identify the visit to the operating room and the corresponding department	Identification of visits to the operating room with the new concept “operating room visit” (VISIT_DETAIL) Relationship between care_site_id of the operating room and care_site_id of the department (CONCEPT_RELATIONSHIP, CONCEPT)
2	Anesthesia procedure during an outpatient visit	Cross-check data from two sources: operating room (AIMS^a^) and hospital stay (PMSI^b^)	Identification of visits to the operating room with the new concept “operating room visit” (VISIT_DETAIL, VISIT_OCCURRENCE)
3	Operations with fast-track surgery and no admission to the PACU^c^	Identify a specific period of the operation	Identification of visits to the operating room with the new concept “operating room visit” (VISIT_DETAIL) Joining with PACU periods (PERIOD)
4	Operations with an MAP^d^<65 mm Hg within 30 minutes of inducing anesthesia	Cross-check data from two secondarily computed, operation-specific periods	Period P1 of hypotension with MAP<65 mm Hg (PERIOD) Period P2 of anesthesia (PERIOD) Joining of P1 and P2 with the start date of P1 in 30 minutes following the start date of P2
5	Administration of norepinephrine, epinephrine, ephedrine, phenylephrine, dobutamine, or atropine received within 15 minutes of the first drop in MAP to below 65 mm Hg	Cross-check data from a secondarily computed period and specific drug administrations	First period P1 of MAP<65 mm Hg (PERIOD) Administration A of norepinephrine, epinephrine, ephedrine, phenyleprine, dobutamine, or atropine (DRUG_EXPOSURE) Linking P1 and A with the start date and time of A in the 15 minutes following the start date of P1 Aggregation by drug
6	Length of stay by ASA^e^ status	Cross-check data from two sources: the operating room (AIMS) and hospital stay (PMSI)	Extraction of ASA status conditions (CONDITION_OCCURRENCE) Linking of the operating room visit details to the visit occurrence (VISIT_OCCURRENCE) Aggregation of the duration of visit occurrence by ASA status
7	Operations followed by a stay in the intensive care unit	Cross-check data from two sources: operating room (AIMS) and hospital stay (PMSI)	Identification of visits to the operating room with the new concept “operating room visit” VD1^f^ (VISIT_DETAIL) Identification of visits to the intensive care unit VD2 (VISIT_DETAIL) Linking VD1 to VD2 according to the visit_occurrence identifier and with VD2 start datetime>VD1 end datatime
8	Characterization of the Mallampati grade	Query the preanesthesia consultation	Extraction of Mallampati scores (NOTE) Aggregation by score

^a^AIMS: anesthesia information management system.

^b^PMSI: Programme de Médicalisation des Systèmes d’Information.

^c^PACU: postanesthesia care unit.

^d^MAP: mean arterial pressure.

^e^ASA: American Society of Anesthesiologists.

^f^VD: visit detail.

**Table 5:** Description of dashboards implemented with the Observational Medical Outcomes Partnership common data model.

Dashboard	Information/objective	Numeric indicators (number or percentage)	Graphics	OMOP^a^ tables
Population description	Overview of the population cared for in the operating room	Number of operations Number of patients Male/female ratio (%) Number of urgent operations (%)	Histogram of age Bar plot of the ASA^b^ Status histogram of the BMI Histogram of the weight	PERSON VISIT_DETAIL OBSERVATION
Hemodynamics	Compliance with hemodynamic guidelines	Number of operations with MAP^c^<65 mm Hg Number of operations with MAP>120 mm Hg Number of operations with HR^d^<60 bpm^e^ Number of operations with SpO2^f^<90%	Bar plot of the duration with MAP<65 mm Hg (min) Bar plot of the duration with MAP>120 mm Hg Bar plot of the duration with HR<60 bpm Bar plot of the duration with SpO2< 90%	PERSON VISIT_DETAIL FEATURE
Ventilation	Compliance with ventilatory guidelines	Number of operations with expiratory tidal volume>8 ml/kg IBW^g^ Number of operations with expiratory tidal volume>10 ml/kg IBW Number of operations with expiratory tidal volume (ml) Number of operations with expiratory tidal volume/IBW (ml/kg)	Bar plot of the expiratory tidal volume>8 ml/kg IBW by sex and year Line plot of the expiratory tidal volume/IBW	PERSON VISIT_DETAIL FEATURE
Postoperative outcome	Overview of postoperative outcome: mortality, duration of hospital stay, and intensive care unit stay	Number of operations followed by a death during hospital stay (%) Duration of hospital stay Number of operations followed by a passage in intensive care (%)	Bar plot of the number of deaths per year Line plot of the number of passages in intensive care per year	PERSON VISIT_DETAIL OBSERVATION

^a^OMOP: Observational Medical Outcomes Partnership.

^b^ASA: American Society of Anesthesiologists.

^c^MAP: Mean arterial pressure.

^d^HR: heart rate.

^e^bpm: beats per minute.

^f^SpO_2_: oxygen saturation.

^g^IBW: ideal body weight.

The OMOP model has a row-oriented structure, with 1 data item per row. For example, each row of OBERVATION stores a single data item (ie, a weight or a BMI). In contrast, each query and dashboard must gather several data items (coming from a single table or several tables). Queries were developed with common table expressions, a syntax provided by PostgreSQL to write auxiliary statements for use in a larger query [43]. Dashboards needed to be implemented on top of the temporary tables gathering the results of a set of CTEs to reduce the response time of each query.

## Discussion

### Principal Results

In the present work, we integrated intraoperative anesthesia data into the OMOP CDM. To the best of our knowledge, this study is the first to have mapped intraoperative data into the OMOP CDM. First, experts from 5 French centers defined a list of concepts describing the anesthesia procedure and specific features. This list mainly comprised standardized concepts from the OHDSI vocabularies: patient history, patient characteristics on the day of the procedure, units, measurements, drugs, and procedure steps. When the corresponding concepts were missing, we added new concepts, particularly to characterize secondarily computed periods and features. Second, we implemented an extract-transform-load process to move perioperative data into the CDM. Third, we implemented common queries related to anesthesia procedures. As the OMOP CDM was initially developed for pharmacoepidemiology, we ensured that the mapping proposed for intraoperative data (and particularly the features specific to our work) could be easily queried. Finally, we developed shareable R scripts for the generation of anesthesia dashboards. These dashboards enabled us to ensure that hemodynamic and ventilatory guidelines were followed.

### Limitations

First, we focused primarily on implementing the vocabulary related to the most common anesthetic procedures. Thus, it may not be sufficient to describe anesthetic management related to more specific procedures (obstetrics, ambulatory procedures, etc), but these could be added in the future. Second, the added concepts are not available in Athena at present and are therefore nonstandard concepts. While waiting for integration validation, the concepts are available on our git directory [44] and can be used and supplemented by other teams. Third, PERIOD and FEATURE are not supported by the OHDSI software stack. Further developments are needed to fully benefit from these new tables in the OHDSI tools and packages. Finally, CDMs may lose information owing to restrictions on the types of relationships proposed in relational models [45]. When integrating, care must be taken to ensure that the information realistically integrated is adequate to perform analyses afterward, and that any loss of information does not sanction the results and their interpretations.

### Comparison With Prior Works

As observed in the studies that focused on specific data (apart from claims data) [29-31], we encountered difficulties with perioperative data. The main difficulty was using several local and custom vocabularies to document the intraoperative period; this contrasts with claims data, which are described according to terminologies. This problem required experts to define anesthesia-related concepts because the local concepts provided by the AIMS were not sufficient. Ryu et al have already reported that mapping by experts is an essential step [46]. Furthermore, the frequency of the recordings (every 30 seconds) in the operating theater produced a large volume of data. We decided not to retain the raw measurements in the measurement table so that the query response time remained acceptable. Raw measurements were stored in a similar measurement table on a twin schema. Finally, we had to compute new periods and features that did not fit in the OMOP CDM tables. To achieve this, we developed 2 new period and feature tables.

Our present work might offer opportunities for research collaborations on intraoperative data with other centers. The material provided here could be used and enhanced by other centers. In combination with federated learning [47], the OMOP CDM provides tools needed for conducting reproducible research.

### Conclusions

Generic data concerning demographics, drugs, units, measurements, and operating room steps were already available in OHDSI vocabularies. However, most of the intraoperative concepts (the duration of specific steps, episodes of hypotension, etc) were absent in the OHDSI vocabularies. We have performed OMOP mapping for reusing anesthesia data.

## Appendix

Multimedia Appendix 1 Anesthesia-related concepts.

Multimedia Appendix 2 Common queries.

## Abbreviations

AIMS: anesthesia information management systems
CDM: common data model
EHR: electronic health record
OHDSI: Observational Health Data Sciences and Informatics
OMOP: Observational Medical Outcomes Partnership
PACU: postanesthesia care unit
PMSI: Programme de Médicalisation des Systèmes d’Information
