# Supplementary material for: Transforming Anesthesia Data Into the Observational Medical Outcomes Partnership Common Data Model: Development and Usability Study
Source: J Med Internet Res. 2021 Oct 29;23(10):e29259. doi: 10.2196/29259 (PMC8590192; doi:10.2196/29259)
Supplement: Multimedia Appendix 2 [file jmir_v23i10e29259_app2.docx]

**Appendix 2 : Common queries**

**Query 1 : Number of operations per year and per speciality department**

**with** operation **as** (

**select** visit_detail_id, **extract**(**year** **from** visit_detail_start_date) **as** **year**, care_site_id

**from** omop.visit_detail vd

**where** vd.visit_detail_concept_id =

(**select** **min**(concept_id) **from** omop.concept **where** concept_name = 'Operating Room Visit')

)

, anesthesia_unit_name **as** (

**select** o.visit_detail_id, **year**, cs.care_site_name **as** anesthesia_unit_name

**from** operation o

**inner** **join** omop.fact_relationship fr

**on** o.care_site_id = fr.fact_id_1

**and** fr.relationship_concept_id = 46233688

**inner** **join** omop.care_site cs

**on** fr.fact_id_2 = cs.care_site_id)

**select** anesthesia_unit_name, **year**, **count**(*) **as** number_operation

**from** anesthesia_unit_name

**group** **by** anesthesia_unit_name, **year**

**order** **by** anesthesia_unit_name, **year**;

**Query 2 : Anesthesia procedures in ambulatory stay**

**select count(*) as ambulatory_stay**

**from** omop.visit_detail vd, omop.visit_occurrence vo

**where** vd.visit_occurrence_id = vo.visit_occurrence_id

-- 'Operating Room Visit'

**and** vd.visit_detail_concept_id = (**select** **min**(concept_id) **from** omop.concept **where** concept_name = 'Operating Room Visit' **and** domain_id = 'Visit')

-- Ambulatory visit

**and vo.visit_start_date = vo.visit_end_date;**

**Query 3 : Operations with fast track surgery without PACU stay**

**select count(*)**

**from** omop.visit_detail vd, omop.visit_occurrence vo

**where** vd.visit_occurrence_id = vo.visit_occurrence_id

**and** vd.visit_detail_concept_id = (**select** **min**(concept_id) **from** omop.concept **where** concept_name = 'Operating Room Visit' **and** domain_id = 'Visit')

**and vd.visit_detail_id not in (select visit_detail_id from feature.period where period_concept_id = 4134563);**

**Query 4 : Operations with a MAP < 65 mmHg within 30 minutes following induction of anesthesia**

**with** hypotension_period **as**

( **select** visit_detail_id, period_start_datetime **as** hypotension_start_datetime

**from** feature.**period** p1

**where** period_concept_id = 2000080170),

anesthesia_period **as**

( **select** visit_detail_id, period_start_datetime **as** anesthesia_start_datetime

**from** feature.**period** p2

**where** period_concept_id = 2000080007)

**select** **count**(**distinct**(t1.visit_detail_id)) **as** nb_operation

**from** hypotension_period t1 **inner** **join** anesthesia_period t2

**on** t1.visit_detail_id = t2.visit_detail_id

**where** t1.hypotension_start_datetime >= t2.anesthesia_start_datetime

**and** t1.hypotension_start_datetime <= (t2.anesthesia_start_datetime + **INTERVAL** '30 minute');

**Query 5 : Administration of norepinephrine, epinephrine, ephedrine, phenyleprine, dobutamine and atropine received within the 15 minutes following the first drop of mean arterial pressure < 65 mmHg**

**with hypotension_period as**

( **select** visit_detail_id, **min**(period_start_datetime) **as** hypotension_start_datetime

**from** feature.**period** p1

**where** period_concept_id = 2000080170

**group** **by** visit_detail_id),

drug_administration **as**

( **select** visit_detail_id, drug_concept_id, drug_exposure_start_datetime

**from** omop.drug_exposure

**where** drug_concept_id **in** (1321341, 1343916, 1143374, 1135766, 1337720, 914335)),

hypotension_drug **as**

( **select** hp.visit_detail_id, da.drug_concept_id

**from** hypotension_period hp **inner** **join** drug_administration da

**on** hp.visit_detail_id = da.visit_detail_id

**and** da.drug_exposure_start_datetime >= hp.hypotension_start_datetime

**and** da.drug_exposure_start_datetime <= (hp.hypotension_start_datetime + **INTERVAL** '15 minute'))

**select** co.concept_name, **count**(*) **as** nb_administration

**from** hypotension_drug hd **inner** **join** omop.concept co

**on** hd.drug_concept_id = co.concept_id

**group** **by** co.concept_name

**order by count(*) desc;**

**Query 6 : Length of stay per ASA Status**

**with** stay **as** (

**select** visit_occurrence_id,

**extract**(**day** **from** visit_end_date::**timestamp** - visit_start_date::**timestamp**) **as** length_stay

**from** omop.visit_occurrence),

asa **as** (

**select** co.visit_occurrence_id, 'ASA' || **right**(c.concept_name,1) **as** asa_status

**from** omop.condition_occurrence co

**inner** **join** omop.concept c

**on** co.condition_concept_id = c.concept_id

**where** co.condition_concept_id **in** (4186042, 4184967, 4186043, 4211334, 4186044, 4186045)

)

**select** asa.asa_status,

**percentile_cont**(0.5) **within** **group**(**order** **by** stay.length_stay) **as** median,

**percentile_cont**(0.25) **within** **group**(**order** **by** stay.length_stay) **as** q1,

**percentile_cont**(0.75) **within** **group**(**order** **by** stay.length_stay) **as** q3

**from** asa **inner** **join** stay

**on** asa.visit_occurrence_id = stay.visit_occurrence_id

**group** **by** asa.asa_status

**order** **by** asa.asa_status;

**Query 7 : Operations followed by a stay in intensive care unit**

**with operation as (**

**select** vd.visit_detail_id, vd.visit_occurrence_id, vd.visit_detail_end_date

**from** omop.visit_detail vd

**where** vd.visit_detail_concept_id = (**select** **min**(concept_id) **from** omop.concept **where** concept_name = 'Operating Room Visit' **and** domain_id = 'Visit')

), intensive_care **as** (

**select** vd.visit_occurrence_id, vd.visit_detail_id, vd.visit_detail_start_date

**from** omop.visit_detail vd

**where** vd.visit_detail_concept_id = 32037

)

**select**

**from** operation o **inner** **join** intensive_care ci

**on** o.visit_occurrence_id = ci.visit_occurrence_id

**and o.visit_detail_end_date <= ci.visit_detail_start_date;**

**Query 8 : Mallampati**

**select n.note_text as mallampati**

, **count**(**distinct** n.visit_detail_id) **as** operation_number

**from** omop.note n

**where** n.note_title = 'MALLAMPATI'

**group by n.note_text;**
